# An experimental study exploring the impact of vignette gender on the quality of university students’ mental health first aid for peers with symptoms of depression

**DOI:** 10.1186/s12889-016-2887-2

**Published:** 2016-02-25

**Authors:** E. Bethan Davies, John Wardlaw, Richard Morriss, Cris Glazebrook

**Affiliations:** Division of Psychiatry and Applied Psychology, School of Medicine, Institute of Mental Health, The University of Nottingham, Triumph Road, Nottingham, NG7 2TU UK; NIHR MindTech Healthcare Technology Co-operative, Institute of Mental Health, The University of Nottingham, Triumph Road, Nottingham, NG7 2TU UK; School of Medicine, The University of Nottingham, Queen’s Medical Centre, Nottingham, NG7 2UH UK

**Keywords:** Mental health literacy, Mental health first aid, Helping behaviours, Depression, University students, Peer support

## Abstract

**Background:**

University students have high rates of depression, and friends are often the most commonly-used source of support for emotional distress in this population. This study aimed to explore students’ ability to provide effective support for their peers with depressive symptoms and the factors influencing the quality of their mental health first aid (MHFA) skills, including students’ gender, course of study, and gender of student experiencing depression.

**Methods:**

Via an online survey, students at two British universities (*N =* 483) were quasi-randomly allocated to view a video vignette of either a male or female student depicting symptoms of depression. An open-ended question probed MHFA actions they would take to help the vignette character, which were rated using a standardised scoring scheme based on MHFA guidelines.

**Results:**

Students reported low MHFA scores (mean 2.89, out of possible 12). The most commonly reported action was provision of support and information, but only eight (1.6 %) students stated an intention to assess risk of harm. Those studying clinically non-relevant degrees with limited mental health content reported poorer MHFA (*p =* <0.001) and were less confident about their ability to support a friend with depression (*p =* 0.04). There was no main effect of vignette gender, but within the group of students on non-relevant courses the male vignette received significantly poorer MHFA than the female vignette (*p =* 0.02). A significant three-way interaction found that male participants studying non-relevant degrees who viewed a male vignette had poorer MHFA compared to females studying non-relevant degrees who viewed the female vignette (*p =* 0.005).

**Conclusions:**

Most students lack the necessary MHFA skills to support friends suffering from symptoms of depression, or to help them get appropriate support and prevent risk of harm. Students on courses which do not include mental health related content are particularly ill-equipped to support male students, with male students receiving the poorest quality MHFA from fellow male students on these courses. MHFA training has the potential to improve outcomes for students with depression, and could have a valuable role in reducing the excess risk of harm seen in male students.

**Electronic supplementary material:**

The online version of this article (doi:10.1186/s12889-016-2887-2) contains supplementary material, which is available to authorized users.

## Background

Depression is one of the most commonly experienced mental health problems in university students. The mean prevalence rate for depression in undergraduate students has been estimated as 30.6 % [1], and there is evidence that students are more at risk of experiencing depression than peers who are not in higher education [2]. Students typically fall within the 18–25 years age bracket: three-quarters of all lifetime cases of mental disorders have their onset by 24 years of age [3]. The mean age of onset and high prevalence rates mean that either students themselves, or one of their friends, is very likely to experience depression.

Worldwide, depression is a leading contributor to disease and cause of disability [4]. Untreated depression can have a significant impact on students’ quality of life, affect their educational experience and the skills they need to complete their degree, and can lead to decreased academic productivity, poorer exam results, absenteeism, social isolation, academic probation and withdrawal from university [5–10]. In students, depression has also been associated with increased risk of developing other mental health problems, increased alcohol consumption, and increased suicidal ideation [11, 12]. Depression may also affect students’ acquisition of professional and interpersonal skills, which subsequently may impact upon their career development. Although studies with young people tend to find that females experience higher rates of common mental health problems [13], the evidence for this in student populations is less conclusive. A number of studies have found no differences in rates of depression between males and females [1]. This may reflect the fact that the stresses of university life, such as exam pressures, are common to both males and females [14]. Furthermore, evidence suggests that male students have poorer outcomes than female peers, and are less likely to get the support they need. Male students report poorer recognition and awareness of mental disorders, greater stigma about mental health and help-seeking, and are less likely to hold favourable attitudes about seeking professional help [15–17]. Young men experiencing depression are also more likely to use harmful coping strategies [18]. Suicide is the leading cause of death for young adults (aged 20–34 years) in England and Wales, with the suicide rate of young men being twice (24 %) that of young women (12 %) [19]. The numbers of university students in England and Wales taking their own lives has risen almost 50 % between 2007 and 2011, with male students making up over two-thirds (69.6 %) of student suicides that occurred in 2011 [20].

Many students who experience clinically-significant depression do not seek out professional help and treatment [21], and increased severity of depression in young people is associated with reduced likelihood of seeking out professional help [22]. Barriers to help seeking in university students include stigma towards mental health problems and help-seeking, lack of knowledge about available help, and not perceiving their mental health problems as sufficiently serious to require help [23, 24]. Some students on vocational courses, such as medicine and nursing, may have additional concerns about how mental health problems may impact on their career, and hold expectations that they should work when unwell [25]. Furthermore, university students often put considerable pressure on themselves and may be reluctant to appear less than perfect to others [26], and the nature of the university environment may influence the level of mental distress considered “normal” and thus influence their perceived need for help [24]. Young people (including university students) often prefer to seek out help for their mental health from their friends, rather than from professionals [22, 27–29]. Friends can play a valuable role in providing early intervention in supporting students’ mental health, as friendships are established, trustworthy, and accessible to students [22, 30]. For example, over 80 % of an Australian student cohort reported seeking help from a close friend for their mental health [29], and qualitative research with British medical students suggested preferences for support from friends rather than from available services [31]. Social networks may also inform young people’s decisions about seeking professional help [27]. Given the prevalence of depression in student populations and their preferences for seeking help from friends, it is important that this population has the skills not only to help themselves, but also to effectively assist a friend experiencing a mental health problem or crisis.

Helping behaviours that a person provides to someone experiencing a mental health problem or crisis are often referred to as mental health first aid (MHFA) [32]. MHFA consists of six actions (abbreviated to ALGEE) applied to someone in need of help: approaching the person; assessing their risk and assisting the person with any crisis; listening non-judgementally; giving support and information; encouraging appropriate professional help; and encouraging other supports [33]. Research with Australian young adults suggests they intend to use and do actually use many MHFA actions when supporting close friends, and perceive them as helpful for the person in need [29, 34–36]. Findings from one Australian study found that although the majority of students (90 %) provided some form of informal help to a friend or relative with a mental health problem, only 24 % stated they encouraged professional help [29]. One barrier to providing effective MHFA may be misconceptions that probing mental health concerns may worsen the friend’s mental health problem or increase their risk of self-harm [37]. Less than a fifth (17 %) of an American student cohort stated they were highly confident in directly asking a friend if they were suicidal [38].

A person’s MHFA skills are underpinned by their mental health literacy [32], which refers to knowledge and attitudes relating to the recognition, treatment and management of mental health problems [39]. There is evidence that mental health literacy varies according to type of degree, with students studying a ‘clinically-relevant’ degree (such as psychology and medicine) having greater awareness of different mental health conditions [40–42]. This may also extend onto students’ MHFA skills.

Greater perceived stigma towards mental health problems [43], being a male providing help [17], and the recipient of help being male [44] have all been shown to be associated with poorer MHFA. As established, male students may be a particularly vulnerable group and vignette methodology, where the participant reads or listens to a third-person account of someone with the symptoms of a mental disorder, offers the opportunity to explore the impact of gender on students’ MHFA provision. [32, 45]. Swami [46] randomised 1218 British participants to receive a text vignette of a male or female character experiencing depression, and found that male participants were less likely to recommend help to the male vignette, compared to the female participants who read the male vignette. It is important to explore whether this interaction is replicated in university students since they tend to rely on peers for help with mental health problems, and male students with depression are particularly vulnerable. Enhancing students’ MHFA skills could serve an important health promotion function by facilitating appropriate help seeking and reducing mental health stigma in this population.

The present study aimed to investigate the quality of university students’ MHFA actions towards a video vignette depicting either a male or female student experiencing symptoms of depression, and their confidence in using those MHFA skills to help a friend. It also aimed to explore factors influencing quality of MHFA, including type of course. A further aim was to explore whether the male vignette receives poorer quality MHFA actions. It was hypothesised that students studying clinically-relevant degrees (i.e. courses with mental health-related curricula) would report better quality MHFA actions and be more confident about providing help to a friend with symptoms of depression.

## Methods

### Design

Participants were quasi-randomised, based on month of birth, to view a male vignette (MV) or a female vignette (FV) depicting a student with symptoms of depression. Allocation was concealed from researchers and participants. The study was administered online through *SurveyMonkey* (Palo Alto, California, USA). The minimum target sample size was 64 in each group, in order to detect 0.5 SD difference in MHFA scores between the male and female vignette groups with *p =* <.05 and 90 % power.

### Ethical approval

This study was reviewed and approved by the University of Nottingham Medical School Research Ethics Committee (reference D11072013IMH). Consent to participate was through ticking a checkbox within the online survey. Prior to giving informed consent, detailed study information was presented on the first page of the online survey.

### Participants and recruitment

Participants were eligible if they were aged ≥18 years and were a registered student at one of two East Midlands universities. Students were invited to take part in a study investigating students’ understanding of psychological distress, which was advertised through circular emails, postings on social media and university intranet, and posters placed around the university. All advertisements signposted towards the URL of online survey. Opportunistic sampling took place in two waves (November-December 2013 and February-April 2014) to avoid exam periods.

### Vignette conditions

For the present study, participants viewed a video clip of either a female student or a male student describing the same symptoms of depression (which aligned with DSM-IV diagnostic criteria) including persistent feelings of sadness, tiredness, and concentration difficulties, and also mentioned parental concern. The script was adapted from previous research [37], and also included some somatic symptoms associated with depression but which may be less known to the general public (see Additional file 1 for vignette script). The two video vignettes were filmed against the same plain background and were of similar duration (78 and 81 s). Identical scripts (except for actor name) were delivered by either a male actor (“Mark”) or a female actor (“Emily”) speaking directly towards the camera without notes/autocue. The two actors were White British, of similar age, and closely matched for appearance and background (e.g. type of clothing, hair colour, tone of voice). Both video clips were reviewed by the third author (RM), a psychiatrist with expertise in mood disorders.

### Measures

#### Socio-demographic variables

Information about participants’ gender (male or female), age, country of origin (home or international student), course of study (presented as a drop-down list of schools/departments) and level of study (undergraduate or postgraduate) was collected.

#### Exposure to mental health issues

Three items ascertained participants’ self-reported experiences of mental health issues: 1) personal experience of mental health issues; 2) close friends and family members affected by mental health issues; and 3) self-reported exposure to media campaigns in the past 12 months. Questions 1 and 2 were assessed through asking participants if either they, or a close friend or family member, had experienced mental health issues similar to those described by the vignette.

### Depressive symptomology

The Patient Health Questionnaire (PHQ-9) [47] screens for probable depression during the previous two weeks. Each item is scored on a four-point scale indicating increased occurrence of the symptom, ranging from ‘not at all’ to ‘nearly every day’. Scores range from 0–27: scores ≤4 imply no depression; 5–9 indicate mild probable depression; 10–14 indicate moderate probable depression; 15–19 indicate moderate-to-severe probable depression; and scores ≥20 suggest severe probable depression [47]. The measure has high sensitivity (0.88) and specificity (0.88) in detecting major depression [47]. Internal consistency for the present study was α = 0.86. The PHQ-9 has been used in student populations and has been previously administered through online surveys [6, 48].

### Stigma towards depression

The Depression Stigma Scale (DSS) [49] consists of two nine-item subscales, which are separately analysed. The personal subscale measures the individual’s personal attitudes towards depression, while the perceived stigma scale assesses the individual’s perceptions about societal attitudes towards depression [49]. Each item is presented with a five-point scale, ranging from ‘strongly disagree’ to ‘strongly agree’. Total scores on each subscale range from 0 to 36, with higher scores indicating greater depression-related stigma. The DSS has been previously administered to adults and adolescents [50–52]. The present study calculated α = 0.81 for the DSS-Personal subscale and α = 0.80 for the DSS-Perceived subscale. To avoid inducing bias in participants’ responses to the open-ended question, the DSS was the final measure administered in the online survey.

### Confidence to help a friend experiencing a mental health problem

Participants self-rated their confidence in helping a friend experiencing symptoms similar to the vignette, from ‘1’ (‘not confident at all’) to ‘4’ (‘very confident’) [35].

### Mental health first aid intentions and confidence

Participants were probed about the actions they would take assuming the vignette was a friend through one open-ended question:*’If Mark/Emily was your friend, what would you do (if anything) to help him/her?’* Participants’ qualitative responses were rated using a standardised scoring scheme developed by experts in MHFA and based upon the adult/standard MHFA action plan [53]. This scheme has been used in previous research exploring MHFA [25, 32, 33, 35, 45, 53, 54]. Responses were scored for each of the six MHFA components: a score of ‘0’ was given if the MHFA component was not mentioned or was an unhelpful response; ‘1’ meant a helpful but superficial response; and ‘2’ meant a good response with specific detail. This produced a score ranging from 0 to 2 for each of the six categorical components, and a total MHFA score ranging from 0 to 12. Higher scores indicate better quality first aid intentions.

### Reliability of coded scores for open-ended MHFA question

To establish inter-rater reliability, EBD (first author) coded 60 open-ended responses from a previous MHFA study [33]. Coded scores were compared to consensus group scores, as coded by the three experts in MHFA [33]. Significant moderate to strong intra class correlations (ICC) were found for the six MHFA components (all *p =* <.001) and for the total MHFA score (r(59) = .87, *p =* <.001). In the present study, all qualitative responses were coded by EBD and JW (second author). A random sample of fifty responses from the present study (reflecting approx. 10 % of the sample) was rated blind by a third rater. Inter-rater reliability was excellent with significant moderate to strong ICCs for the six MHFA components (all *p =* <.001) and for the total MHFA score (r(49) = .81, *p <* .001).

### Procedure

The study was administered through an online survey: prior to recruitment, the online survey was quality checked (e.g. testing of quasi-randomisation procedure and accessibility of video vignettes) through piloting with eight volunteers. The online survey took approximately 20 min to complete. Participants provided demographic information before being randomised to view either the MV or FV based on month of birth (January = MV, February = FV, March = MV, etc.). After viewing the vignette, participants completed the outcome measures. On completing the study, they were debriefed and informed that the video vignettes were actors depicting symptoms typical of young people’s experience of depression. At this stage they were also provided with contact details for local support services for mental health problems, and given the opportunity to enter a prize-draw to win a voucher.

### Statistical analysis

Data were analysed using SPSS V.21 (Chicago, IL, USA). Factors relating to participants’ demographic background, their degree of study, the vignette character they saw, their mental health, and personal and perceived stigma towards depression were included in analyses relating to their level of MHFA. Parametric (ANOVA) and non-parametric analyses (Chi-Square tests, Mann Whitney U tests) were used depending on the normality of the data’s distribution, and p-values ≤ .05 were considered statistically significant. Any missing data for each variable were excluded crosswise in analyses: overall there was less than 1 % of data missing.

## Results

**Fig. 1:**
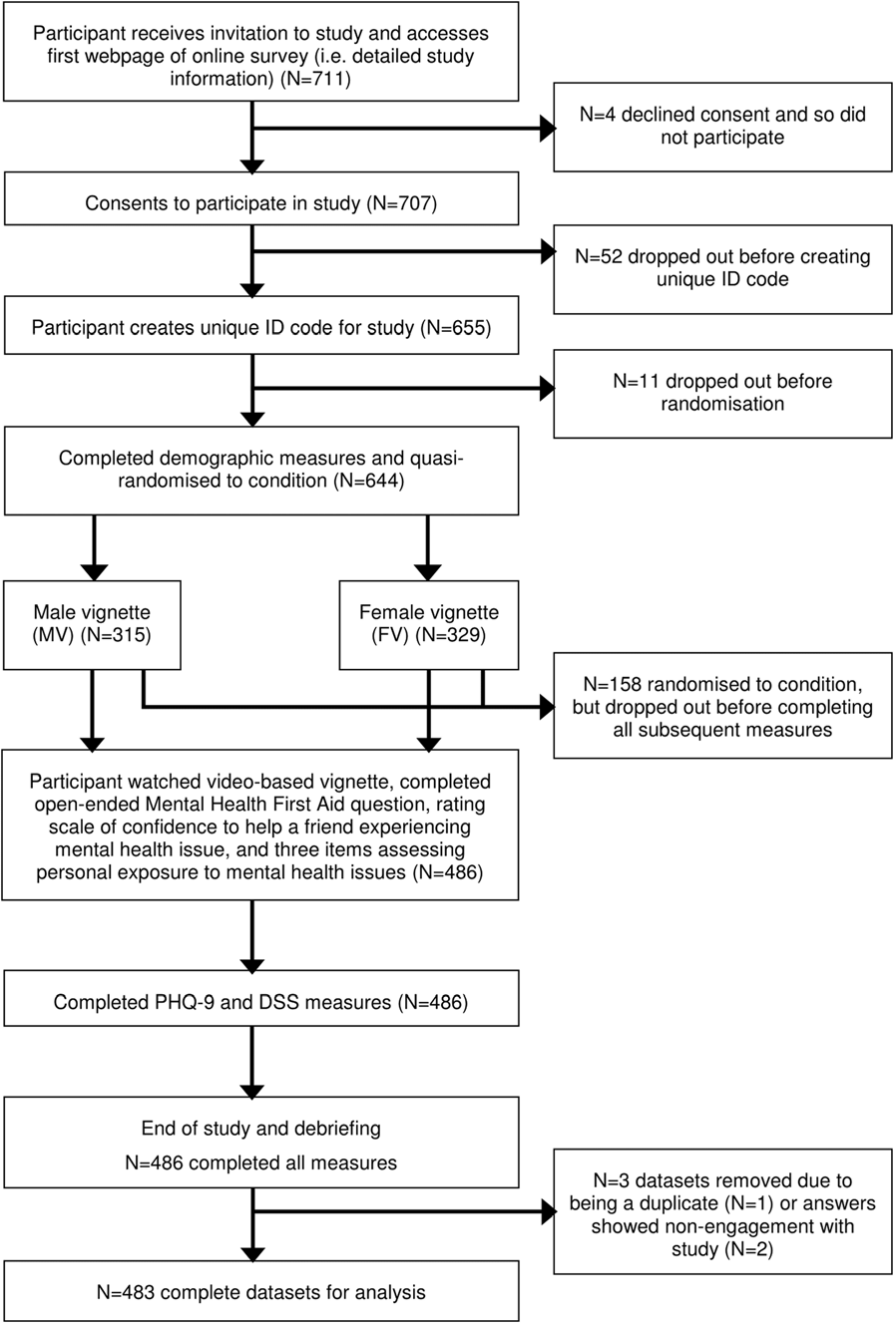
Participant flow through study. NB: FV = Female vignette; MV = Male vignette

A total of 707 students consented to participate, with 221 (31.3 %) failing to complete the study. Figure 1 shows participant flow throughout the study. Analysis showed no socio-demographic (participant gender, age, clinical relevancy of degree, year of study, level of study, and country of origin) or allocation (MV or FV) differences between non-completers and completers. After removing the non-completers, 483 participants were included in analysis. The sample’s mean age was 21.6 (±4.76) years, with a median age of 20 years. Over half of the sample (*N =* 273, 56.5 %) stated they had experienced a similar issue to the vignette or another psychological issue, with 29.6 % (*N =* 143) meeting the moderate, moderate-to-severe, or severe depression thresholds on the PHQ-9.

The two vignette groups were well matched on demographic characteristics except for year of study: more first year students saw the FV, and more second year students saw the MV (*X*^2^(3) = 13.93, *p =* .003) (Table 1). Participants studying degrees with mental health content (medicine, nursing, psychology, sociology and social work) were coded as studying clinically-relevant degrees (*N =* 161, 33.3 % of sample). All other degree subjects were collapsed into a non-relevant degrees category (*N =* 320, 66.3 %). Through a Chi Square test, there were more males than expected by chance who were not studying a clinically-relevant degree, and fewer males than expected studying a relevant degree. The opposite occurred for females: there were fewer females than expected by chance who were not studying a clinically-relevant degree, and more females than expected studying a relevant degree (*X*^2^ (1) = 9.298, *p =* .002).

**Table 1:** Demographic characteristics of the sample, separated by vignette condition

	All (*N =* 483)	Participant saw FV (*N =* 244)	Participant saw MV (*N =* 239)
	N (%)	N (%)	N (%)
Age (mean, SD)	21.62 (±4.76)	21.42 (±3.85)	21.82 (±5.54)
Gender			
Male	126 (26.1)	57 (23.4)	69 (28.9)
Female	357 (73.9)	187 (76.6)	170 (71.1)
Type of degree			
“Clinically-relevant” degree	161 (33.3)	85 (34.8)	76 (31.8)
“Non-relevant” degree	320 (66.3)	158 (64.8)	162 (67.8)
Level of education			
Undergraduate	436 (90.3)	217 (88.9)	219 (91.6)
Postgraduate	46 (9.5)	26 (10.7)	20 (8.4)
Origin			
Home (UK) student	393 (81.4)	197 (80.7)	196 (82)
EU or International student	88 (18.2)	46 (18.9)	42 (17.6)
Depressive symptomology (mean, SD)	7.25 (±6.21)	6.92 (±5.68)	7.59 (±6.70)
Threshold level of depression			
None	207 (42.9)	101 (41.4)	106 (44.4)
Mild	133 (27.5)	78 (32)	55 (23)
Moderate	72 (14.9)	34 (13.9)	38 (15.9)
Moderate-to-severe	41 (8.5)	22 (9)	19 (7.9)
Severe	30 (6.2)	9 (3.7)	21 (8.8)
Personal experience of mental health issue(s)			
Yes	273 (56.5)	137 (56.1)	136 (56.9)
No	180 (37.3)	93 (38.1)	87 (36.4)
Experience of family member/close friend with mental health issue(s)			
Yes	295 (61.1)	148 (60.7)	147 (61.5)
No	129 (26.7)	68 (27.9)	61 (25.5)
Exposure to mental health media/campaigns in past twelve months			
Yes	280 (58)	143 (58.6)	137 (57.3)
No	121 (25.1)	60 (24.6)	61 (25.5)
Personal stigma towards depression (mean, SD)	8.84 (±5.65)	9.31 (±5.65)	8.36 (±5.61)
Perceived public stigma towards depression (mean, SD)	20.67 (±5.34)	20.52 (±5.09)	20.83 (±5.59)

NB: FV = Female vignette; MV = Male vignette. Numbers do not add up to 100 % for some factors due to missing data

### Differences between participants studying clinically-relevant and non-relevant degrees

Compared to those studying a clinically-relevant degree, participants studying a non-relevant degree had significantly higher depressive symptomology scores (7.56 ± 6.17 vs. 6.54 ± 6.07 in those studying clinically-relevant degrees; Z = 2.04, *p =* .041), had less confidence in helping a friend similar to the vignette (2.60 ± 0.81 vs. 2.77 ± 0.76 in clinically-relevant degrees; Z = −2.06, *p =* .040), and held greater personal stigmatising attitudes towards depression (9.76 ± 5.72 vs. 6.91 ± 4.94 in clinically-relevant degrees; Z = −5.30, *p =* <.001). There were no differences between the two groups in level of perceived public stigma.

### Quality and predictors of MHFA actions

The median number of MHFA actions reported was two (range 0 to 5) out of a possible six. The most endorsed actions were providing support and information (*N =* 307, 63.5 %), followed by encouraging professional help (*N =* 283, 58.6 %) and listening non-judgementally (*N =* 258, 53.5 %). Only eight students (1.6 %) identified the need to assist with a potential crisis or assess the vignette character’s risk of harm. Female participants were more likely to suggest providing support and information (66.4 % of females vs. 55.6 % males: *X*^2^(1) = 4.717, *p =* .030). Studying a clinically-relevant degree was significantly associated with suggesting listening non-judgementally (65.2 % vs. 47.5 % in non-relevant degrees: *X*^2^(1) = 13.51, *p =* <.001), and encouraging other supports (39.8 % vs. 27.5 %: *X*^2^(1) = 7.43, *p =* .006), but not encouraging professional help (*X*^2^(1) = 3.55, *p =* .059). Additional file 2 shows the total percentage of participants mentioning the six MHFA components and the scores assigned to each component.

Total MHFA scores (assessing the overall quality of MHFA actions) in the sample ranged from 0 to 9 (out of 12), with a mean of 2.89 (±1.52). Being female (Z = −2.30, *p =* .021), studying a clinically-relevant degree (Z = −5.536, *p =* <.001), being a home student (Z = −3.28, *p =* .001), having experience of a family member/close friend with a mental health issue (Z = −3.89, *p =* <.001), recent exposure to mental health media/campaigns (Z = −3.017, *p =* .003), being older (r_s_(481) = .125, *p =* .006), and lower levels of personal stigma (r_s_(483) = −.252, *p =* <.001) were statistically associated with higher MHFA scores. Depressive symptomology, level of education, personal experience of mental health problems and perceptions of societal stigma were unrelated to MHFA scores (all p= > .05), and there was no statistical difference between those who saw the FV (2.99 ± 1.54) and those who saw the MV (2.78 ± 1.49); Z = 1.56, p= > .05. Table 2 displays the mean MHFA scores for each variable.

**Table 2:** Associations between total MHFA scores and categorical demographic factors

Variable	Mean MHFA score (SD)	Statistic	P-value
Gender		Z = −2.30	.021
Male (*N =* 126)	2.67 (1.54)		
Female (*N =* 357)	2.96 (1.50)		
Type of degree		Z = −5.54	<.001
‘Clinically-relevant' (*N =* 161)	3.45 (1.67)		
‘Non-relevant' (*N =* 320)	2.60 (1.36)		
Level of education		Z = −0.45	.652
Undergraduate (*N =* 436)	2.89 (1.56)		
Postgraduate (*N =* 46)	2.84 (1.09)		
Origin		Z = −3.28	.001
Home student (*N =* 393)	2.99 (1.54)		
EU/International student (*N =* 88)	2.43 (1.32)		
Personal experience of mental health issue(s)		Z = −1.50	.652
Yes (*N =* 273)	3.02 (1.57)		
No (*N =* 180)	2.76 (1.43)		
Experience of family member/close friend with mental health issue(s)		Z = −3.89	<.001
Yes (*N =* 295)	3.13 (1.58)		
No (*N =* 129)	2.49 (1.30)		
Exposure to mental health media/campaigns in past twelve months		Z = −3.02	.003
Yes (*N =* 280)	3.08 (1.50)		
No (*N =* 121)	2.66 (1.57)		

### The impact of vignette gender on MHFA scores

**Fig. 2:**
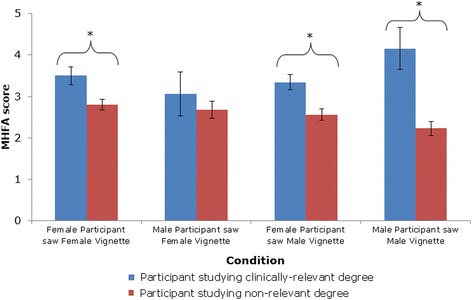
Mean MHFA scores for each condition, sub-grouped by whether participant was studying a clinically-relevant degree. Error bars indicate Standard Deviations. Bars marked with asterisks (*) indicate a significant difference in MHFA scores between clinically-relevant and non-relevant degree participants within the Participant Gender x Vignette Gender condition

A three-way ANOVA was performed with participant gender (male/female), vignette gender (male/female) and type of degree (clinically-relevant/non relevant) as the independent factors, and total MHFA score as the dependent variable. A main effect was found for type of degree, F (1, 473) = 28.51, *p =* <.001: those studying a clinically-relevant degree had higher MHFA scores. There were no main effects for participant gender or vignette gender. There was a statistically significant interaction between vignette gender and type of degree, F(1, 473) = 5.25, *p =* .022. For participants studying clinically-relevant degrees, there were no differences in MHFA scores between the MV (M = 3.48 ± 1.52) and FV conditions (M = 3.42 ± 1.80: Z = −2.55, p= > .05). However, for those studying non-relevant degrees, those who viewed the MV had lower MHFA scores (M = 2.45 ± 1.36), indicating poorer quality help, compared to those studying non-relevant degrees who viewed the FV (2.77 ± 1.33: Z = −2.37, *p =* .018). There was also a three–way interaction between participant gender, vignette gender and type of degree F(1, 473) = 4.192, *p =* .041). Post-hoc analyses found that male participants studying non-relevant degrees who saw the MV had significantly lower scores (M = 2.23 ± 1.26) than female participants studying non-relevant degrees who saw the FV (M = 2.80 ± 1.35: Z = −2.837, *p =* .005). There were no significant vignette gender and participant gender differences in those studying clinically-relevant degrees (Fig. 2).

### Self-rated confidence in helping a friend with depressive symptoms

Only 63 (13 %) were very confident that they could help a friend, with 186 (38.5 %) stating were only slightly confident or not confident at all. Students studying a non-relevant degree reported lower self-confidence (2.60 ± 0.81) than those studying clinically-relevant degrees (2.77 ± 0.76; Z = −2.06, *p =* .04). In the clinically-relevant and non-relevant degree groups respectively, there were no differences between males and females in level of confidence (all p= > .05). There were no participant gender or vignette gender differences in level of perceived confidence regardless of type of degree. Higher total MHFA scores (r_s_(483) = 0.23, *p =* <.001), higher depression scores (r_s_(483) = .09, *p =* .039) and lower personal stigma (r(483) = −.186, *p =* <.001) were all associated with greater level of perceived confidence to help a friend.

## Discussion

The overall quality of proposed actions to help a friend with symptoms of depression was generally poor, as indicated by a mean MHFA score of 2.89 out of a possible 12. Although the majority of students (64 %) would provide support and information to a fellow student, less than 2 % would assess risk of harm. Furthermore, only 13 % of students felt very confident about helping a friend with symptoms of depression and nearly 40 % expressed no confidence or only slight confidence. As predicted, students undertaking courses with mental health-related curricula had higher MHFA scores and increased self-confidence to help a friend, suggesting that they were better placed to help other students who had mental health issues. In the group as a whole, females had better mental health first aid skills than males, probably reflecting the greater proportion of female students studying clinically-relevant degrees. Lower personal stigma and greater experience of mental health issues through family and friends and media campaigns were also associated with higher MHFA scores. The prevalence of depression in this study (29 %) was typical of student samples [1], but depression scores and personal experience of mental health problems were unrelated to MHFA skills, suggesting that those experiencing symptoms of depression may not be particularly well placed to help themselves or others. Vignette gender did not impact on MHFA skills in the sample as a whole, but within the group of students studying non-relevant degrees the male vignette received poorer quality MHFA. Furthermore, a significant three-way interaction suggested male participants studying non-relevant degrees were less equipped to provide MHFA to male students with symptoms of depression, compared to female participants faced with a female student with the same symptoms.

To our knowledge, this is the first study to have used the developed MHFA coding scheme in the UK, and has shown how students’ MHFA skills can vary by their course of study, their own gender and gender of the person they may be helping. The total MHFA score for those studying clinically-relevant degrees in the present study aligns with scores found in Australian medicine and nursing students [25]. Mental health literacy is likely to underpin students’ MHFA skills, resulting in more and better quality helping intentions in students on courses with clinically-relevant content. The particularly poor MHFA scores for males studying non-relevant courses is a concern. Although a systematic review showed that the majority of studies found lower rates of depression in male students (25 %) compared to female students (29 %), the difference in rates is small and some studies reported statistically similar rates of depression for males and females [1]. What is clear, however, is that young males are more likely to delay or avoid mental health help-seeking and are at more risk for attempting suicide [18]. A systematic review of prevalence rates of depression in university students found a mean weighted prevalence rate of 25.6 % (95 % CI, 23.2–26.6) for the 10 studies sampling medical students, compared to 35.6 % (95 % CI, 34.9–37.8) in studies which sampled a range of courses [1]. Therefore students studying non-relevant degrees may be more vulnerable to depression, be less able to recognise a potential mental health problem, and have peers who are less able to provide good quality support.

The Australian research team behind much of the research into MHFA have developed a professionally-led face-to-face group-based training course (‘Standard MHFA’) [55]. This course is delivered by a trained instructor and is designed to educate trainees about how to recognise a mental health problem (both in themselves and in others), the types of treatment and self-help available for mental health, and how to help an individual experiencing a mental health crisis, or experiencing the early developmental stages of a mental health problem. This course has been adapted for adults and young people working with different populations [33]. The course has been modified to be delivered via online e-learning, which is completed by participants at their own pace, and does not require a trained instructor or any face-to-face group sessions [25, 56]. ASIST (Applied Suicide Intervention Skills Training) is another similar course [57]. Unlike Standard MHFA, ASIST appears to focus on one mental health-related issue; ASIST is designed to increase trainees’ knowledge about suicide and providing first aid to someone who is suicidal, whereas Standard MHFA addresses a range of mental health issues and crises. A recent meta-analysis suggests the professionally-led MHFA training courses are effective in improving knowledge and attitudes relating to mental health problems, and improve the supportive behaviours provided to someone with a mental health problem [58]. The Standard MHFA course is considered to be a cost-effective intervention, given that it is delivered in groups by experienced trainers, and trains individuals to have empowerment over their own and others’ mental health [59]. Through delivering this course to students, it could help foster a community who can effectively support their own mental health and the mental health of other students.

Recent research suggests that both online and face-to-face MHFA training for nursing and medical students improves the quality of their intentions to help someone with depression and confidence in their ability to provide help, as well as helping reduce mental health-related stigma [25]. Online and technological interventions for improving common mental health problems have shown promising results in student populations [60, 61]. As universities often use web-based platforms (e.g. virtual learning environments, such as Moodle) in delivering higher education, students are likely to be highly familiar with online e-learning and using multimedia educational content, suggesting the e-learning MHFA course could be a particularly useful intervention for university students. Randomised trials of interventions to improve students’ mental health literacy and MHFA are needed to evaluate the impact of MHFA training on the actual quality of peer support, utilisation of healthcare services, and mental health outcomes.

### Strengths and limitations

In previous research exploring the public’s MHFA actions for another person, vignettes are often either read by the participant or read aloud by the researcher via a telephone interview [34, 44, 54]. This type of vignette may provide a less realistic presentation of a mental health problem, and video-based vignettes have been suggested as portraying a more realistic presentation of depression [62]. Using video vignettes was a novel and engaging way of showing a hypothetical person experiencing depression, and the study’s online delivery provided students with some flexibility in participating in the study. A potential weakness of using video vignettes is that there may be unanticipated differences between the two vignettes’ presentation which are not attributable to gender (e.g. perceived severity of the symptoms). However the lack of overall differences in MHFA by vignette gender suggests that the vignettes were well matched. Future research may want to explore potential differences that could arise in using text-based and video-based vignettes in research exploring the public’s MHFA skills, such as how the type of vignette delivery may influence reported MHFA actions.

A strength of the study is the standardised assessment of quality of MHFA which followed the gold standard method of scoring as used in previous studies [25, 32, 33, 35, 45, 53, 54]. The open-ended MHFA question allowed participants to provide their own unprompted responses, and provides a better representation of knowledge than closed questions [63]. This also allows participants to produce their own perspectives, rather than rating researcher-defined helping behaviours [64]. Inter-rater reliability was limited to only one external rater but did involve approx. 10 % of the sample’s responses, which is a higher percentage than seen in similar research. Furthermore, agreement between raters was excellent.

As in previous student-based research [1], male participants were under-represented in this sample and this may have limited the study’s power to fully explore the interaction between vignette gender and participant gender. The sample had an over-representation of female students, British (home) students, and undergraduates, meaning the findings may not be generalizable to the wider student population within the UK. The study used a convenience sample and those with a personal interest in mental health or had experience of mental health problems may have been more likely to participate. However the thresholds found in the sample’s depressive symptomology scores were typical for a student population.

Noticeably, almost a third (*N =* 221) of those who consented failed to complete the study, which could potentially have influenced the findings. Data provided by the online survey suggested that most of these drop outs withdrew from the study on the webpage where they had to watch the video. This may have been the deciding factor in fully participating in the study – those who dropped out may have been put off by watching a video, or were not in a suitable environment to play the video. Furthermore, technical problems and accessibility issues may have impacted upon participants’ decision and ability to view the video vignettes and continue their participation in the study, although we did not detect any technical difficulties in pilot testing of the online survey. There were no socio-demographic differences between completers and non-completers. The use of month of birth to allocate to condition is not truly random and may have introduced unanticipated bias, but there was no evidence of this.

Finally the present study did not ask whether participants had previously attended the MHFA course, and only assessed participants’ MHFA intentions to a hypothetical student experiencing depression and not their actual MHFA behaviours. However an Australian vignette study using the same MHFA coding scheme as the present study found that MHFA intentions were predictive of actual MHFA behaviours [54].

## Conclusions

The results of this study suggest students are not well-equipped to provide MHFA to a fellow student with depression, particularly if they are studying courses without mental health content. There is evidence that males with depression may be disadvantaged. The poorest quality MFHA was observed in male students in non-relevant degrees presented with a male student with symptoms of depression. As university students often seek out help from their friends for their mental health, it is important for this population to have sufficient MHFA skills and confidence to be able to support a friend in need. An ability to assess and manage risk of harm could be crucial, but only a tiny proportion of students had this skill. MHFA training has the potential to improve outcomes for students with depression and could have a valuable role in reducing the excess risk of harm particularly seen in male students.

## Additional files

Additional file 1: Script spoken by both vignettes in the videos. (PDF 41.6 kb) Additional file 2: Descriptive data showing the coded scores for each MHFA action, subgrouped by 1) participant gender X vignette gender condition; 2) participant gender; and 3) type of degree studied. (PDF 19.3 kb)

## Abbreviations

DSS: Depression stigma scale
FV: Female vignette
MHFA: Mental health first aid
MV: Male vignette
PHQ-9: Patient health questionnaire – 9 item version
